# Comparative dosimetric assessment of combined treatment modalities in cervical cancer radiotherapy for optimal organ protection

**DOI:** 10.1007/s00411-025-01113-7

**Published:** 2025-03-01

**Authors:** Iozsef Gazsi, Loredana G. Marcu

**Affiliations:** 1 Faculty of Physics, West University of Timisoara, 300223 Timisoara, Romania; 2 Emergency County Hospital, 410167 Oradea, Romania; 3 UniSA Allied Health & Human Performance, University of South Australia, Adelaide, SA 5001 Australia; 4 Faculty of Informatics & Science, University of Oradea, 410087 Oradea, Romania

**Keywords:** 3D-conformal radiotherapy, Intensity modulated radiotherapy, Volumetric modulated arc therapy, Brachytherapy, Organs at risk, Combined radiotherapy, Dosimetry

## Abstract

External radiotherapy combined with internal radiotherapy in cervical cancer can provide a boost to the target volume to increase tumour control. At the same time internal radiotherapy protects neighboring organs. The aim of the present study was to dosimetrically compare three external beam radiotherapy techniques each combined with internal radiotherapy to evaluate the combination that offers the best organ protection. Treatment plans of 20 cervical cancer patients were created for external (including three-dimensional conformal radiotherapy (3D-CRT), intensity modulated radiotherapy (IMRT) and volumetric modulated arc therapy (VMAT)) as well as brachytherapy. The prescribed dose was 50 Gy in 25 fractions for external and 21 Gy in three fractions for internal radiotherapy. The following organs at risk (OARs) were evaluated: bladder, rectum, sigmoid and bowel bag. The study analyzed the results of different treatment combinations in terms of dosimetric values for various parameters. The D_90_ for the clinical target volume was around 120 Gy, with the highest value seen in 3D-CRT + BT (brachytherapy) combination at 120.59 Gy. For the bladder, the D_2cc_ remained below the recommended threshold of 90 Gy, with the lowest value obtained for the BT + IMRT combination at 79.2 Gy. For the rectum, both D_2cc_ and D_1cc_ remained below the recommended threshold of 75 Gy for both parameters. All techniques fell below the recommended dose of 75 Gy for the sigmoid. For the intestine, there were statistically significant differences between BT + IMRT and BT + 3D-CRT. The VMAT technique showed superiority over IMRT in tumour volume coverage and several organ-at-risk parameters. Generally, intensity-modulated techniques showed dosimetric advantage over the traditional 3D technique in cervical cancer. In addition to providing better compliance and homogeneity, they provided superior protection for organs at risk, especially for bowel bag. It is concluded that the BT + IMRT technique provided the best protection for organs at risk based on the lowest OAR dosimetric values, especially for the intestine.

## Introduction

Cervical cancer is the third most common cancer in Romania and the second most common cause of death among women (Bruni et al. 2023). The five-year survival rate after treatment for some types of cancer in Romania is much lower than the European Union average. In addition, 7.5% of all cervical cancer cases diagnosed in Europe come from Romania (Bruni et al. 2023). The treatment of cervical cancer depends on the staging of the tumour and the patient’s condition, and can include surgery, chemotherapy and radiotherapy (Bruni et al. 2023).

External beam radiotherapy (EBRT) with concomitant chemotherapy, followed by brachytherapy, is the standard treatment for locally advanced cervical carcinoma (WHO 2023).

External radiotherapy can be delivered using a variety of methods, including 3D conformal radiotherapy (3D-CRT), intensity modulated radiotherapy (IMRT), and volumetric arc-modulated treatment (VMAT). 3D-CRT uses shaped beams to precisely target tumours, minimizing damage to surrounding tissue. IMRT delivers radiation of varying intensity from multiple angles, allowing for precise dose modulation. Finally, VMAT combines intensity modulation and rotational delivery. In VMAT, the radiation source continuously rotates around the patient, delivering radiation in a dynamic arc while modulating the intensity and shape of the beam. However, most radiotherapy centers in Romania continue to use 3D-CRT for the treatment of cervical cancer (Gazsi and Marcu 2023).

Brachytherapy is an essential component of cervical irradiation, allowing a very high local dose to be delivered to the target region. High Dose Rate (HDR) brachytherapy has essentially superseded Low Dose Rate (LDR) brachytherapy due to its distinct advantages of compact source and applicator size, fast treatment periods, and improved control of source location and dose distribution. Iridium-192 (Ir^192^) is the most commonly used radioisotope in HDR brachytherapy and delivers a localized and highly targeted dose of radiation to the tumour, sparing surrounding healthy tissue. This technique allows a high dose of radiation to be delivered directly to the tumour, increasing effectiveness while reducing side effects. The specific type and duration of brachytherapy treatment depends on several factors, including the type and stage of the cancer. When it comes to HDR brachytherapy, the Fletcher applicator is the most often utilized (Mourya et al. 2021).

Considering that two different treatment procedures are applied (an external and an internal radiotherapy technique) it is critical to check the total dose received by the tumour volume and the organs at risk (OARs). The combination of the two techniques can deliver a significantly higher dose to the tumour volume than either one of them, while still offering a good protection to the surrounding organs.

In Romania, most public hospitals employ the 3D-CRT technique as standard of care for cervical cancer, despite the known advantage of intensity modulated techniques for other pathologies (Grégoire et al. 2015; Burela et al. 2019). Therefore, the present study aims to analyze the results of combined treatment techniques, for a cohort of 20 patients diagnosed with cervical cancer treated at the County Emergency Clinical Hospital in Oradea, Romania, with 3D-CRT and brachytherapy for which IMRT and VMAT plans have been simulated for dosimetric comparative purpose. The goal was to evaluate the superiority (if any) of combined intensity modulated techniques with brachytherapy versus conformal radiotherapy + brachytherapy in terms of both target and normal tissue dosimetry.

## Materials and methods

### Patient selection and planning objectives

This study included 20 patients treated at the County Emergency Clinical Hospital in Oradea, Romania, between 2021 and March 2023. All patients had a histologically proven diagnosis of cervical cancer with stages between IIA and IVA. CT scans were performed with a thickness of 5 mm between slices from the lumbar spine 1 to the proximal third of the femoral shaft. Patients consumed 500 ml of water half an hour before the scan for the bladder to have the same volume both during the CT scan and during each treatment fraction. Patients were placed on the treatment couch in supine position with their hands above their heads, according to the treatment protocol.

Target volumes and structures at risk (rectum, bladder, bowel bag, sigmoid and femoral heads) were delineated according to Reports 62 and 83 of the International Commission on Radiation Units and Measurements (ICRU 1999, 2010). The cervix, pelvic nodes and parametrial tissues were all part of the clinical target volume (CTV). The CTV was included in the planning target volume (PTV), with 5 mm margins considered in all directions. On all CT slices for all patients, both target volumes and OARs were outlined.

The dosimetric prescription for external beam radiotherapy to the PTV was 50 Gy in 25 fractions of 2 Gy per fraction. The minimum constraint for the PTV specified that at least 95% of the prescribed dose should be delivered to 95% of the volume. The maximum dose was also set at no more than 110%, but preferably around 105%. The dose constraints were the same for all external techniques used, bladder V_40_ < 60% (less than 60% of the bladder volume should receive 40 Gy), rectum V_50_ < 50%, bowel bag V_45_ < 10% (less than 10% of the bowel bag volume should receive 45 Gy) or V_45_ < 195 cm^3^ (less than 195 cm^3^ of the bowel bag volume should receive 45 Gy) and femoral heads D_max_ < 42 Gy. In addition to dose constraints, other quantities such as PTV_105_, D_2cc_, D_98_, D_90_, V_10_, V_20_ etc. were also examined for a more accurate comparison.

For brachytherapy, the prescribed dose was 21 Gy in three fractions. For the bladder, the limitation requires that more than 2 cm^3^ of the organ should receive less than 80% of the prescribed dose; for the rectum, bowel and sigmoid, the limitation requires that more than 2 cm^3^ of the organ should receive less than 70% of the prescribed dose. The equivalent dose was calculated based on radiobiological considerations, and summed with the dose provided by the external radiotherapy techniques. The results were analyzed according to ICRP 89 recommendations and compared with data reported in the scientific literature (ICRU 2013).

#### Planning techniques for external radiotherapy

The Monaco 6.1.2.0 planning system of the Elekta Synergy Linac with a 0.5 cm multi-leaf collimator (MLC), a total of 160 leaves, with a maximum field size of 40 × 40 cm^2^ was used to create all treatment plans, including 3D-CRT, IMRT and VMAT.

The anterior, posterior, left and right lateral four-field “box” approach with 15 MV photon beam was used to create the 3D-CRT plans. The field-in-field technique was employed to reduce the hot spots after the MLC leaves were conformed to the PTV by 5 mm.

For IMRT, treatment plans were created using the dMLC feature of the Monaco system, which provides step and shoot in addition to variable dose rate and the ability to dynamically move the leaves.

For VMAT plans with 6 MV photons, two arcs—one clockwise and one counterclockwise—were used, starting at an angle of 180° and collimating at 5° to minimize lamellar transmission. These arcs conformed to the PTV by 5 mm.

#### Planning techniques for brachytherapy

The Fletcher applicator with two ovoids was used to optimize the brachytherapy treatment plan, offering the possibility to irradiate the cervix with the upper part of the vagina. To reduce the discomfort of applicator insertion, patients were placed in the lithotomy position and given local anesthesia. Based on patients’ anatomy the depth and angle of the applicator was individually determined, with three standard angles being available (30°, 45°, and 60°). The size of the ovules was also established (2 cm, 2.5 cm, or 3 cm). After insertion of the intrauterine applicator, the two ovules were placed at the beginning of the cervix.

Following the CT scan at the planning station, a virtual applicator was placed over the real one to replicate the doses received by the tumour volume and OARs. The radioactive source was placed in an applicator hole at five-millimeter intervals. These positions were used to calculate the time the source remains stationary to cover the tumour volume and protect nearby organs while achieving the appropriate dose distribution. For treatment plan evaluation, the tumour volume, named CTV_HR_, and the surrounding OARs were outlined.

The applicator was selected from the planning library and manually overlaid on the applicator images captured on CT, followed by the manual placement of source positions. Between eight and 16 source positions were employed, depending on the size and length of the tumour volume. Treatment plans were manually optimized by adjusting the dwell time of the source in each position until optimal coverage of the tumour volume, protection of OARs, and a pear-shaped isodose were achieved.

The resulting isodose is pear-shaped, due to the intrauterine applicator located in the cervix and the two ovoids located in the upper perimeter of the vagina. High radio-toxicity may occur in the bowel, but the bladder and rectum are generally well protected.

Throughout the literature, it is shown that many institutions use various HDR brachytherapy fractionation regimens, including 5.5 Gy × 5 fractions, 6 Gy × 4 fractions, 7 Gy × 3 fractions, and 9 Gy × 2 fractions (ICRU 2010). The protocol used in the present study involved 21 Gy in 3 fractions.

### Dosimetric evaluation tools

Quantitative evaluation of the treatment plans was performed using the mean and maximum values of delineated volumes of interest using the standard dose-volume histogram (DVH). Dosimetric parameters for PTV and OARs were compared between 3D-CRT-IMRT and IMRT-VMAT. The percentages of PTV receiving 95%, 105%, 107% and 110% of the prescribed dose (PTV_95_, PTV_105_, PTV_107_ and PTV_110_) were considered. The dosimetric comparison of OARs between the techniques is presented by the mean doses, by the volume percentage to receive 10 Gy, 20 Gy, 30 Gy, 40 Gy, 45 Gy and 50 Gy (V_10_, V_20_, V_30_, V_40_, V_45_ and V_50_) and the D_2cc_, D_1cc_ and D_0.1 cc_, i.e., the dose to 2 cm^3^, 1 cm^3^ and 0.1 cm^3^, respectively, of the specific organ.

The conformity index (CI) and homogeneity index (HI) were calculated according to Eq. 1:

1 $$CI = \frac{{\mathop V\nolimits_{RX}^{2} }}{{TV*\mathop V\nolimits_{RI} }},HI = \frac{{\mathop D\nolimits_{5\% } }}{{\mathop D\nolimits_{95\% } }}$$

where for CI the V_RX_ is the volume of the structure covered by the dose of interest, TV is the total volume of the structure and V_RI_ is the total volume of the isodose of interest; for HI the D_5%_ and D_95%_ are the minimum doses in 5% and 95% of the PTV volume, respectively, that received the prescribed dose. CI and HI values closer to 1 indicate better dose conformity and homogeneity of the plan.

### Calculation of the equivalent dose for brachytherapy and summing with EBRT

EQD2 (dose equivalent for 2 Gy) is the dose normalized to 2 Gy per fraction, where the effect of the initial dose is equal to the effect of treating the tumour volume with 2 Gy per fraction.

The formula used to calculate the normalized dose is (Eq. 2):

2 $${EQD}_{2}=D*([d+(\frac{\alpha }{\beta })]/[2 Gy+\left(\frac{\alpha }{\beta }\right)])$$

where D is the total dose delivered, d is the dose per fraction, and α/β is the dose at which the linear and quadratic components of cell killing on the cell survival curve are equal. The parameters α and β indicate the intrinsic radio resistance of the tumour or healthy organ. The α/β factor was used with a value of 10 (Gy_10_) for tumour volume and α/β with a value of 3 (Gy_3_) for organs at risk (Fowler 1989; Hall 2019; van Leeuwen et al. 2018).

By summing the EBRT (D_EBRT_) and brachytherapy (D_BT_) doses, the total dose was determined for each volume of interest (Eq. 3):

3 $$\mathop D\nolimits_{Total} = \mathop D\nolimits_{EBRT} + \mathop D\nolimits_{BT}$$

For the combined dose, the target values for tumour volume were D_98_ and D_90_, which are also the most commonly reported in the literature. The OARs assessed were bladder, rectum, sigmoid, and bowel bag, for which the parameters D_2cc_, D_1cc_, and D_0.1 cc_ were analyzed.

### Statistical analysis

A paired Student t-test was used to evaluate the statistical significance of the dosimetric differences between the treatment techniques. The threshold for statistical significance was set at p < 0.05.

## Results

Table 1 shows the results obtained for all data ± standard deviation for PTV. Table 2 presents the dosimetric parameters of OARs focusing on bladder, rectum, bowel bag and femoral heads. In addition, both tables provide data for a comparison between 3D-CRT and IMRT or VMAT with p-value, while the quality indices include the conformity index and the homogeneity index.

**Table 1:** Mean values and the standard deviations of the evaluated dosimetric parameters for planning target volume (PTV) according to all three external beam radiation therapy (EBRT) techniques (three-dimensional conformal radiotherapy (3D-CRT), intensity modulated radiotherapy (IMRT) and volumetric modulated arc therapy (VMAT)); Statistically significant values are bolded (p < 0.05); CI—conformity index; HI—homogeneity index; PTV_xx_—percentage of PTV receiving xx% of the prescribed dose; D_xx_—the dose received by xx% of the PTV; D_max_—the maximum dose received by the PTV

PTV parameter	3D-CRT	IMRT	VMAT	3D-CRT vs. IMRT	IMRT vs. VMAT
Volume (cm^3^)	990.65 ± 672.19	990.3 ± 671.84	990.25 ± 671.79	–	–
PTV_95_ (Gy)	48.7 ± 1.07	48.69 ± 0.61	49.06 ± 1.43	0.89	**0.05**
PTV_105_ (%)	0.03 ± 0.13	0.01 ± 0.02	0.02 ± 0.1	0.19	**0.02**
PTV_107_ (%)	0 ± 0	0 ± 0	0 ± 0	–	–
PTV_110_ (%)	0 ± 0	0 ± 0	0 ± 0	–	–
D_98_ (Gy)	46.8 ± 3.14	48.48 ± 0.97	48.57 ± 1.84	** < 0.001**	0.06
D_90_ (Gy)	49.76 ± 1.17	49.06 ± 0.73	49.35 ± 1.18	** < 0.001**	0.09
D_max_ (Gy)	52.4 ± 0.99	52.72 ± 0.6	53.17 ± 0.58	** < 0.001**	** < 0.001**
CI	0.61 ± 0.34	0.84 ± 0.15	0.8 ± 0.1	**0.02**	**0.03**
HI	1.06 ± 0.02	1.05 ± 0.01	1.04 ± 0.03	** < 0.001**	0.07

**Table 2:** Mean values and the standard deviations of the evaluated dosimetric parameters for organs at risk (OARs) according to all external beam radiation therapy (EBRT) techniques (three-dimensional conformal radiotherapy (3D-CRT), intensity modulated radiotherapy (IMRT) and volumetric modulated arc therapy (VMAT)); statistically significant values are bolded (p < 0.05); D_mean_—the mean dose received by the organ at risk; D_xxcc_—dose received by xx cm^3^ volume of the organ at risk; V_xx_ (%)– the volume in % receiving at least xx Gy; V_xx_ (cm^3^)—the volume in cm^3^ receiving at least xx Gy; D_max_—the maximum dose received by the organ at risk

Organ at risk	Parameter	3D-CRT	IMRT	VMAT	3D-CRT vs. IMRT	IMRT vs. VMAT
Bladder	Volume (cm^3^)	134.76 ± 180.11	140.19 ± 174.685	140.18 ± 174.69	–	–
	D_Mean_ (Gy)	40.63 ± 9.1	38.23 ± 1.62	36.06 ± 4.4	** < 0.001**	** < 0.001**
	D_2cc_ (Gy)	51.31 ± 3.3	49.42 ± 1.16	50.12 ± 2.17	** < 0.001**	** < 0.001**
	D_1cc_ (Gy)	51.41 ± 1.91	49.64 ± 1.09	50.36 ± 2.14	** < 0.001**	**0.003**
	D_0.1 cc_ (Gy)	51.66 ± 1.48	50.21 ± 1.11	50.93 ± 2.13	** < 0.001**	**0.002**
	V_10_ (%)	100 ± 0	100 ± 0	99.82 ± 3.53	**–**	0.31
	V_20_ (%)	100 ± 0	97.15 ± 6.83	88.83 ± 11.85	** < 0.001**	** < 0.001**
	V_30_ (%)	96.92 ± 25.88	79.16 ± 10.02	67.19 ± 20.63	** < 0.001**	** < 0.001**
	V_40_ (%)	51.6 ± 32.74	48.15 ± 14.32	45.57 ± 13.45	0.27	0.21
	V_45_ (%)	39.57 ± 37.94	31.36 ± 17.53	31.88 ± 12.74	**0.02**	0.85
	V_50_ (%)	20.6 ± 46.19	0.58 ± 1.04	5.5 ± 9.68	** < 0.001**	** < 0.001**
Rectum	Volume (cm^3^)	75.2 ± 158.57	78.56 ± 155.21	78.55 ± 155.22	–	–
	D_Mean_ (Gy)	39.14 ± 8.49	38.21 ± 5.21	35.73 ± 5.61	0.56	**0.01**
	D_2cc_ (Gy)	50.25 ± 6.81	48.49 ± 1.56	49.14 ± 2.2	** < 0.001**	**0.04**
	D_1cc_ (Gy)	50.66 ± 5.74	48.83 ± 1.41	49.64 ± 1.76	** < 0.001**	**0.002**
	D_0.1 cc_ (Gy)	51.33 ± 4.06	49.37 ± 2.36	50.48 ± 1.27	** < 0.001**	** < 0.001**
	V_10_ (%)	95.87 ± 32.45	95.54 ± 15.75	95.04 ± 16.91	0.55	0.82
	V_20_ (%)	96.7 ± 13.19	93.17 ± 15.92	90.59 ± 15.82	0.66	0.29
	V_30_ (%)	87.69 ± 40.1	86.24 ± 22.77	72.93 ± 19.96	0.83	** < 0.001**
	V_40_ (%)	49.8 ± 40.47	53.6 ± 24.56	43.58 ± 24.59	0.37	**0.01**
	V_45_ (%)	35.13 ± 40.89	29.57 ± 18.73	25.74 ± 20.71	0.32	0.27
	V_50_ (%)	15.41 ± 29.83	0.16 ± 0.85	1.72 ± 8.25	** < 0.001**	** < 0.001**
Bowel bag	Volume (cm^3^)	1144.58 ± 5063.43	1147.71 ± 5059.67	1147.74 ± 5059.64	–	–
	D_Mean_ (Gy)	24 ± 9.62	22.97 ± 5.72	23.57 ± 10.09	0.41	0.61
	D_2cc_ (Gy)	50.38 ± 6.02	47.02 ± 3.75	48.09 ± 2.81	** < 0.001**	**0.05**
	D_1cc_ (Gy)	50.74 ± 4.39	47.66 ± 3.28	48.67 ± 2.48	** < 0.001**	**0.03**
	D_0.1 cc_ (Gy)	51.29 ± 2.05	48.85 ± 2.42	49.82 ± 2.16	** < 0.001**	** < 0.001**
	V_10_ (%)	80.49 ± 23.96	86.01 ± 15.74	88.15 ± 19.3	0.09	0.42
	V_20_ (%)	64 ± 25.33	58.55 ± 21.56	59.57 ± 30.68	0.19	0.83
	V_30_ (%)	36.66 ± 27.17	25.75 ± 19.86	29.02 ± 42.72	**0.01**	0.41
	V_40_ (%)	10.05 ± 22.42	6.45 ± 7.86	8.37 ± 18.75	0.09	0.25
	V_45_ (%)	6.46 ± 15.06	2.01 ± 6.88	2.56 ± 4.46	** < 0.001**	0.38
	V_45_ (cm^3^)	79.6 ± 269.69	34.89 ± 228.8	31.54 ± 152.1	**0.01**	0.83
	V_50_ (%)	3.1 ± 13	0.02 ± 0.28	0.04 ± 0.15	** < 0.001**	0.57
Sigmoid	D_2cc_ (Gy)	49.15 ± 2.3	46.68 ± 3.57	48.38 ± 6.53	0.06	**0.05**
	D_1cc_ (Gy)	49.87 ± 2.14	47.58 ± 3.19	49.22 ± 3.84	**0.02**	**0.009**
	D_0.1 cc_ (Gy)	50.69 ± 1.73	48.85 ± 2.86	50.18 ± 2.24	**0.004**	**0.002**
Right femoral head	Volume (cm^3^)	108.33 ± 56.67	110.69 ± 59.09	110.67 ± 59.07	–	–
	V_30_ (%)	5.14 ± 30.03	7.98 ± 8.59	10.26 ± 14.37	0.29	0.44
	V_40_ (%)	0 ± 0.02	0.01 ± 0.05	0.01 ± 0.02	**0.02**	0.66
	D_max_ (Gy)	34.65 ± 6.94	38.74 ± 7.12	40.11 ± 8.77	** < 0.001**	**0.1**
Left femoral head	Volume (cm^3^)	109.83 ± 61.18	112.27 ± 63.64	112.28 ± 63.65	–	–
	V_30_ (%)	9.79 ± 69.85	6.92 ± 8.56	9.82 ± 12.17	0.62	0.13
	V_40_ (%)	0 ± 0	0.01 ± 0.05	0.02 ± 0.09	**0.02**	0.41
	D_max_ (Gy)	33.96 ± 7.92	38.78 ± 10.2	39.94 ± 9.32	** < 0.001**	0.23

### PTV dosimetry

All three techniques achieved more than 95% coverage of the tumor volume, with VMAT achieving the best coverage at 49.06 Gy (98.12%). Volume over 105% was lowest for IMRT with 0.01% and for VMAT with 0.02% with a statistically significant difference (p = 0.02). For D_98_, statistically significant differences of 1.68 Gy (p < 0.001), were observed between 3D-CRT and IMRT, whereas for D_90_, the significant difference is in favor of 3D-CRT with a dose difference of 0.7 Gy (p < 0.001). The maximum dose was found to be lowest for 3D-CRT (52.4 Gy) and highest for VMAT (53.17 Gy), all with statistically significant differences (p < 0.001). IMRT conformity index (0.84) was the best compared to VMAT (0.8) and 3D-CRT (0.61), with statistically significant differences. The homogeneity index for VMAT was found to be closest to 1 with a value of 1.04.

### OARs dosimetry

For the bladder, statistically significant differences were observed between 3D-CRT and IMRT for almost all parameters, the most significant being V_30_ with differences of 17.76% and V_50_ with 20.02%, all in favor of IMRT. Comparing IMRT and VMAT techniques, the most significant difference can be observed for V_50_ with 4.92% in favor of IMRT. For the parameters D_2cc_, D_1cc_, D_0.1 cc_ the highest values obtained were for 3D-CRT with more than 51 Gy and the lowest for IMRT.

The lowest mean dose to the rectum was obtained with the VMAT technique (35.73 Gy) compared to IMRT (38.21 Gy) (p = 0.01). For D_2cc_, D_1cc_ and D_0.1 cc_, the lowest values were achieved with IMRT and the highest with 3D-CRT, with statistically significant differences. For both V_30_ and V_40_, statistically significant differences were observed comparing IMRT and VMAT (p < 0.01). For V_50_, IMRT technique obtained the lowest values compared to 3D-CRT or VMAT (p < 0.001).

**Fig. 1:**
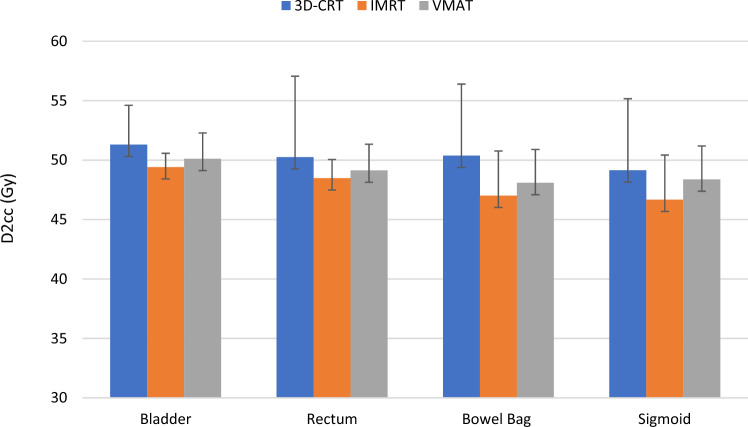
D_2cc_ parameter compared between the three techniques (three-dimensional conformal radiotherapy (3D-CRT), intensity modulated radiotherapy (IMRT) and volumetric modulated arc therapy (VMAT)) for bladder, rectum, bowel bag and sigmoid (see Table 2). The error bars represent the standard deviation of the data relative to the mean

The bowel bag received the lowest mean dose with IMRT: 22.97 Gy compared to 24 Gy with 3D-CRT and 23.57 Gy with VMAT. For D_2cc_, D_1cc_, D_0.1 cc_, IMRT also achieved the lowest values with significant differences compared to the other two techniques as seen in Fig. 1. Statistically significant differences were observed between 3D-CRT and IMRT for parameters V_30_, V_45_, V_45(cm3)_ and V_50_ in favor of IMRT.

For the sigmoid, the lowest values were obtained with the IMRT technique for all parameters, with statistically significant differences for D_1cc_ and D_0.1 cc_ when comparing 3D-CRT with IMRT and IMRT with VMAT techniques.

For femoral heads, the 3D-CRT technique obtained the lowest values for D_max_ with statistically significant differences compared to the other two techniques. For V_40_, significant differences were observed between 3D-CRT and IMRT. The maximum dose from IMRT was 38.74 Gy administered to the left femoral head and 38.78 Gy to the right femoral head. However, 3D-CRT planning achieved better protection of both femoral heads with 4.09 Gy and 4.82 Gy, respectively, lower doses than from IMRT.

### External treatment combined with brachytherapy

In the present study the results from treatment combinations (EBRT + brachytherapy) were further analysed in terms of dosimetric values for various parameters, using the calculated EQD doses (Eq. 2). The D_90_ for CTV (clinical target volume) was about 120 Gy_10_, with the highest value seen in the 3D-CRT + BT (brachytherapy) combination at 120.59 Gy_10_ (Table 3). All combinations met the ICRU 89 recommendation of 85–90 Gy_10_. The highest value for D_98_ was observed in the BT + VMAT combination at 105.62 Gy_10_, with all combinations achieving values above 103 Gy_10_.

**Table 3:** Mean values of dosimetric parameters calculated from the radiobiological summation of external radiotherapy (three-dimensional conformal radiotherapy (3D-CRT), intensity modulated radiotherapy (IMRT) and volumetric modulated arc therapy (VMAT)) and brachytherapy (BT) doses (Eqs. 2 and 3). CTV_HR_—CTV high risk; Gy_3_—indicates the biological dose considering α/β = 3 for the organs at risk; Gy_10_ indicates the biological dose considering α/β = 10 for the tumour; D_xx_ – the dose received by xx% of the CTV_HR_;D_xxcc_—dose received by xx cm^3^ volume of the organ at risk

		ICRU (2013)	**BT + 3D-CRT**	**BT + IMRT**	**BT + VMAT**	**3D-CRT vs. IMRT**	**IMRT vs. VMAT**
			D	D	D	P value	P value
CTV_HR_ (Gy_10_)	D_98_	–	103.87 ± 6.52	105.26 ± 7.96	105.62 ± 7.11	0.25	0.74
	D_90_	85–90	120.59 ± 7.5	119.88 ± 8.05	120.15 ± 7.33	0.56	0.82
Bladder (Gy_3_)	D_0.1 cc_	–	93.74 ± 7.11	92.27 ± 6.16	92.98 ± 6.13	0.2	0.53
	D_1cc_	–	85.21 ± 6.62	83.41 ± 6.06	84.13 ± 5.68	0.11	0.51
	D_2cc_	< 90	81.13 ± 7.11	79.2 ± 6.76	79.9 ± 6.23	0.09	0.52
Rectum (Gy_3_)	D_0.1 cc_	–	79.92 ± 10.37	77.95 ± 8.16	79.07 ± 7.66	0.22	0.46
	D_1cc_	–	74.59 ± 8.5	72.76 ± 8.28	73.56 ± 7.83	0.18	0.53
	D_2cc_	< 75	72.16 ± 8.69	70.4 ± 9.81	71.03 ± 9.55	0.26	0.67
Sigmoid (Gy_3_)	D_0.1 cc_	–	61.49 ± 2.25	59.65 ± 8.57	60.98 ± 5.58	0.32	0.53
	D_1cc_	–	58.37 ± 5.58	56.08 ± 8.12	57.72 ± 7.85	0.11	0.25
	D_2cc_	< 75	56.15 ± 7.55	53.68 ± 6.14	55.38 ± 6.65	0.08	0.09
Bowel bag (Gy_3_)	D_0.1 cc_	–	73.43 ± 8.61	70.98 ± 7.33	71.92 ± 6.21	0.08	0.53
	D_1cc_	–	68.18 ± 6.46	65.1 ± 6.01	66.06 ± 7.48	**0.007**	0.41
	D_2cc_	< 75	64.44 ± 4.6	61.08 ± 8.49	62.09 ± 9.85	** < 0.001**	0.31

For the bladder, D_2cc_ remained below the recommended threshold of 90 Gy_3_, with the lowest value achieved with the BT + IMRT combination at 79.2 Gy_3_. The highest value achieved with BT + 3D-CRT did not exceed 82 Gy_3_. Similarly, the values for D_1cc_ were also below 90 Gy_3_, with the highest value for BT + 3D-CRT at 85.21 Gy_3_. For D_0.1 cc_ the combined plans achieved values below 94 Gy_3_, with the highest value observed for BT + 3D-CRT at 93.74 Gy_3_.

For the rectum, both D_2cc_ and D_1cc_ remained below the recommended threshold of 75 Gy_3_. The highest values were seen in the BT + 3D-CRT combination with D_2cc_ at 72.16 Gy_3_ and D_1cc_ at 74.59 Gy_3_. The highest value for D_0.1 cc_ was also observed for BT + 3D-CRT with 79.92 Gy_3_, while the lowest was observed for BT + IMRT with 77.95 Gy_3_.

All techniques offered dosimetric values below the recommended dose of 75 Gy_3_ to the sigmoid. However, the combination of BT + IMRT resulted in the lowest values for all parameters, with doses of 53.68 Gy_3_ for D_2cc_, 56.08 Gy_3_ for D_1cc_, and 59.65 Gy_3_ for D_0.1 cc_.

For the bowel, there were statistically significant differences between techniques. BT + IMRT achieved the lowest dosimetric values with 61.08 Gy_3_ for D_2cc_ and 65.1 Gy_3_ for D_1cc_ compared to BT + 3D-CRT with 64.44 Gy_3_ for D_2cc_ and 68.18 Gy_3_ for D_1cc_.

**Fig. 2:**
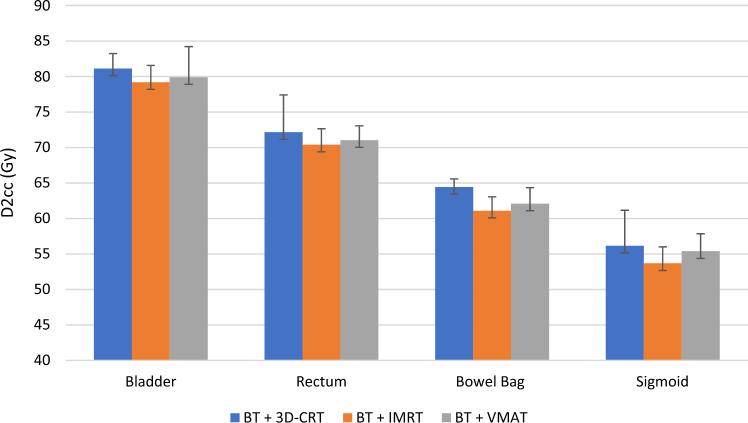
D_2cc_ parameter compared between the three techniques (three-dimensional conformal radiotherapy (3D-CRT), intensity modulated radiotherapy (IMRT) and volumetric modulated arc therapy (VMAT)) summed including brachytherapy (BT) for bladder, rectum, bowel bag and sigmoid (see Table 3). The error bars represent the standard deviation of the data relative to the mean

As can be seen in Fig. 2, the initial differences between the three types of treatment after brachytherapy are minimal for most OARs.

## Discussion

The aim of this study was to compare dosimetric data obtained from treatment plans for three EBRT techniques and, based on radiobiological considerations on cell killing and cell survival, to sum them with data from BT for dosimetric assessment of tumour volume and OARs, in order to evaluate the optimal combinations for the management of cervical cancer.

As indicated by the results, both modulated intensity techniques (VMAT, IMRT) show certain dosimetric advantages to 3D-CRT in terms of target coverage and dose to OARs alike, providing better conformity and homogeneity: When comparing the two intensity modulated techniques, VMAT was superior for tumour volume coverage, while IMRT provided better dosimetry for organs at risk according to several evaluated parameters. Studies comparing IMRT and VMAT in cervical cancer have shown comparable target coverage and sparing of critical organs (Barbera et al. 2023). However, VMAT often offers advantages in terms of treatment efficiency and reduced treatment time, which can improve patient comfort and accessibility. Additionally, shortening the treatment time can also lead to reduced intra-fraction uncertainties (Li et al. 2013). IMRT, on the other hand, offers precise control over beam intensity and direction, potentially optimizing dose distribution in complex cases (Pötter et al. 2018).

Although the 3D-CRT technique provides high tumour control, it has been shown to be less protective of OARs. This is also due to the lower number of photon fields involved in planning, which results in poorer conformation to the tumour volume, with quadratic isodoses not conforming to the tumour volume contour. On the other hand, this conformation helps to protect the femoral heads, since these OARs can be completely covered by the collimator blades from two angles, resulting in a lower maximum dose. Thus, the lowest values with statistically significant differences were obtained with this technique compared to the modulated intensity techniques.

For the present study, a systematic review of the literature was performed to compare the data obtained with the literature. The analysis of the published data indicated that the tumour volume achieved good coverage with good conformation. Bandyopadhyay et al. specified that patients who received a mean dose higher than 79.75 Gy_10_ had significantly better tumour control compared to those who were below this dose (87.1% vs. 52.9%) (Bandyopadhyay et al. 2021).

The ICRU 89 recommendation whereby D_90_ should be 85–90 Gy_10_ was achieved by Kumar et al. (Kumar et al. 2019a, b) with 89.03 Gy_10_, by Pötter et al. (2011) with 93 Gy_10_, and by Kirisits et al. (Kirisits et al. 2005) with 87 Gy_10_ (Table 4). Additionally, Li et al. (Li et al. 2022) reported 79.1 Gy_10_ while Tang et al. (Tang et al. 2020) delivered 83.7 Gy_10_ with high local control. Compared with the results of the present work, all values reported in the literature were lower, i.e., in the present study the tumour volume was covered with a higher mean dose. It is noted that D_98_ was only reported by Bandyopadhyay et al. with a value of 69.29 Gy_10_ (Bandyopadhyay et al. 2021).

**Table 4:** Dose regimens administered for cervical cancer patients, and tumour volume dosimetry according to published literature and compared to the results of the present study. External beam radiation therapy (ERBT), three-dimensional conformal radiotherapy (3D-CRT), intensity modulated radiotherapy (IMRT) and volumetric modulated arc therapy (VMAT); D_90_—dose received by the 90% of the CTV high risk; CTV_HR_—CTV high risk; EQD_2_—equivalent dose in 2 Gy; N/A—not available

Study / nr of patients	EBRT technique and dosage	Brachytherapy dose	D_90_ CTV_HR_ EQD_2_ (Gy_10_)
Present study	3D-CRT 50 Gy/25 fractions	7 Gy/3 fractions	120.59 ± 7.5
	IMRT 50 Gy/25 fractions		119.88 ± 8.05
	VMAT 50 Gy/25 fractions		120.15 ± 7.33
Bandyopadhyay et al. 2021 48 patients	3D-CRT 50 Gy/25 fractions	7 Gy/3 fractions	82.46 ± 6.58
Kumar et al. 2019a 35 patients	3D-CRT 46 Gy/23 fractions	9 Gy/2 fractions	N/A
Okonogi et al. 2020 36 patients	3D-CRT 50 Gy/25 fractions	7 Gy/ 2 fractions	70.0
Kumar et al. 2019b N/A patients	3D-CRT 45 Gy/25 fractions r	7 Gy/4 fractions	89.03 ± 2
		9 Gy/ 2 fractions	69.16 ± 4
Pötter et al. 2011 156 patients	3D-CRT 45–50.4 Gy	7 Gy/4 fractions	93 ± 13
Jamalludin et al. 2015 N/A patients	3D-CRT 48.6 Gy/27 fractions	7 Gy/4 fractions	80.8
Kirisits et al. 2005 22 patients	3D-CRT 45 Gy/25 fractions	7 Gy/4 fractions	87 ± 10
Beriwal et al. 2011 44 patients	3D-CRT/IMRT 45 Gy/25 fractions	5–6 Gy/4 fractions	83.3
Tharavichitkul et al. 2013 47 patients	3D-CRT/IMRT 45 Gy/25 fractions	6.5–7 Gy/4 fractions	93.1 ± 7.7
Nomden et al. 2013 46 patients	IMRT 45 Gy/25 fractions	7 Gy/4 fractions	84

For bladder D_2cc_ doses, the highest value was 88.5 Gy_3_ reported by Okonogi et al. (Okonogi et al. 2020), while the lowest value was 68.58 Gy_3_ reported by Kumar et al. (2019a, b) (Table 5). In comparison, the present study obtained the lowest value of 79.2 Gy_3_. For the rectum, most studies followed the ICRU recommended dose of 75 Gy_3_, except for Okonogi et al. (2020), who achieved 79.2 Gy_3_ for D_2cc_. The lowest value was 57.5 Gy_3_ found by Beriwal et al. (Beriwal et al. 2011), while the present study achieved 70.4 Gy_3_. Regarding the D_1cc_ and D_0.1 cc_ doses, the reported values varied among the studies.

**Table 5:** Dosimetry of organs at risk (OARs) according to published literature and compared to the results of the present study. Three-dimensional conformal radiotherapy (3D-CRT); intensity modulated radiotherapy (IMRT); brachytherapy (BT); D_xxcc_—dose received by the xx cm^3^ of the organ at risk; Gy_3_—indicates the biological dose considering α/β = 3 for the organs at risk; EQD_2_ – equivalent dose in 2 Gy; N/A – not available. Note: in the study by Kumar et al. (2019a) the two sets of data are presented for (1) general anesthesia (GA) and (2) procedural sedation (PS), while in Kumar et al. (2019b) the two sets of data originate from two distinct fractionation schedules used for BT (7 Gy × 4 fractions vs 9 Gy × 2 fractions)

Study [ref]	Bladder D_2cc_ EQD_2_ (Gy_3_)	Bladder D_0.1 cc_ EQD_2_ (Gy_3_)	Rectum D_2cc_ EQD_2_ (Gy_3_)	Rectum D_0.1 cc_ EQD_2_ (Gy_3_)	Sigmoid D_2cc_ EQD_2_ (Gy_3_)	Sigmoid D_0.1 cc_ EQD_2_ (Gy_3_)
Present study results for BT + 3D-CRT	81.13 ± 7.11	93.74 ± 7.11	72.16 ± 8.69	79.92 ± 10.37	56.15 ± 7.55	61.49 ± 2.25
Present study results for BT + IMRT	79.2 ± 6.76	92.27 ± 6.16	70.4 ± 9.81	77.95 ± 8.16	53.68 ± 6.14	59.65 ± 8.57
Present study results for BT + VMAT	79.9 ± 6.23	92.98 ± 6.13	71.03 ± 9.55	79.07 ± 7.66	55.38 ± 6.65	60.98 ± 5.58
Bandyopadhyay et al. 2021 BT + 3D-CRT	81.30 ± 10.34	100.22 ± 12.39	69.31 ± 5.75	82.53 ± 10.79	64.28 ± 12.39	N/A
Kumar et al. 2019a BT + 3D-CRT	GA- 79.51	GA- 105.16	GA- 63.47	GA-72.60	GA-62.20	GA-75.43
	PS-77.96	PS- 100.23	PS-69.54	PS- 83.60	PS-62.91	PS-74.91
Okonogi et al. 2020 BT + 3D-CRT	88.5	N/A	79.2	N/A	69.1	N/A
Kumar et al. 2019b BT + 3D-CRT	77.26 ± 9 (BT—7 Gy/4fractions)	93.22 ± 15 (BT—7 Gy/4fractions)	71.11 ± 6 (BT—7 Gy/4fractions)	88.65 ± 12 (BT—7 Gy/4fractions)	55.44 ± 7 (BT—7 Gy/4fractions)	66.30 ± 14 (BT—7 Gy/4fractions)
	68.58 ± 7 (BT -9 Gy/2fractions)	75.20 ± 12 (BT -9 Gy/2fractions)	64.82 ± 5 (BT -9 Gy/2fractions)	79.59 ± 11 (BT -9 Gy/2fractions)	52.14 ± 7 (BT -9 Gy/2fractions)	60.60 ± 12 (BT -9 Gy/2fractions)
Pötter et al. 2011 BT + 3D-CRT	86 ± 17	N/A	65 ± 9	N/A	64 ± 9	N/A
Jamalludin et al. 2015 BT + 3D-CRT	81.7	N/A	73.9	N/A	N/A	N/A
Kirisits et al. 2005 BT + 3D-CRT	83 ± 9	121 ± 25	64 ± 6	77 ± 10	63 ± 7	79 ± 12
Beriwal et al. 2011 BT + 3D-CRT/IMRT	79.7	N/A	57.5	N/A	66.8	N/A
Tharavichitkul et al. 2013 BT + 3D-CRT/IMRT	88.2 ± 7.2	N/A	69.6 ± 6.6	N/A	72 ± 6.9	N/A
Nomden et al. 2013 BT + IMRT	83	107	66	80	61	71

The articles reviewed showed that the D_2cc_ doses to sigmoid were always below the recommended value of 75 Gy_3_. (Table 5). In the present study, BT + IMRT achieved a value of 53.68 Gy_3_, while Kumar et al. (Kumar et al. 2019a, b) obtained 52.14 Gy_3_ with brachytherapy administered with a 9 Gy/2 fraction regimen. The D_1cc_ value of 67 Gy_3_ was reported by Kirisits et al. (2005), compared to 56.08 Gy_3_ in the present study. Other studies presented for D_0.1 cc_ 60.6 Gy_3_ (Kumar et al. 2019a, b) and 71 Gy_3_ (Nomden et al. 2013). In the present work, all values for D_0.1 cc_ were below 62 Gy_3_ for all three modalities. Bowel values were only reported by Nomden et al. (2013) with D_2cc_ to be 64 Gy_3_, while the present study reported 61.08 Gy_3_ for BT + IMRT. For D_0.1 cc_, Nomden et al. (2013) obtained 77 Gy_3_ compared to 70.98 Gy_3_ in the present study. The highest value found in the present study for BT + 3D-CRT was 73.43 Gy_3_.

Many studies including that by Tanderup et al. (2016) reported higher tumour control for doses above 85 Gy_10_ for D_90_. Mazeron et al. (2016) reported high grade 3 toxicity for patients receiving doses above 75 Gy_3_ for D_2cc_ and Georg et al. (2011) reported a higher probability of grade 3 toxicity for patients with doses above 88 Gy_3_ and 76 Gy_3_ for D_0.1 cc_ and D_2cc_, respectively. In a retrospective study conducted by Manir et al. (2016), doses between 64 Gy_3_ and 69 Gy_3_ for D_2cc_ and between 75 Gy_3_ and 81 Gy_3_ for D_0.1 cc_ were recommended, to avoid grade 3 rectal toxicity. Mazeron et al. (2016) concluded that D_2cc_ < 65 Gy may be associated with less rectal toxicity, whereas D_2cc_ > 75 Gy may predict higher toxicity and also more frequent rectal morbidity. In the present study, doses around 71 Gy_3_ for D_2cc_ and 79 Gy_3_ for D_0.1 cc_ were observed, which are in line with the literature recommendations.

While in the present study OARs were protected by the investigated techniques, the EMBRACE II trial suggested that higher D90 (> 95 Gy) doses to the tumour volume did not achieve significant local control rate improvements, but instead increased the dose of OARs, aggravating their side effects (Pötter et al. 2018).

Although some dosimetric differences are significant both between intensity modulated techniques and 3D-CRT and also among IMRT and VMAT, the additional doses from brachytherapy reduced these differences, and for some parameters, such as D_2cc_, they were even no longer statistically significant.

While the local control rate of cervical cancer has increased significantly with image-guided brachytherapy (IGBT), but the regional control rate has not improved (Nomden et al. 2013; Castelnau-Marchand et al. 2015). A clinical cut-off value of 87 Gy was recently proposed for D_90_ in HR-CTV for the combined EBRT + IGBT treatment of cervical tumours (Dimopoulos et al. 2009a). It was showed that compared to patients receiving D_90_ < 87 Gy, those receiving doses higher than 87 Gy (4%) experienced local recurrence (Dimopoulos et al. 2009b). Wu et al. (Wu et al. 2024) concluded that local tumour control can be achieved for doses between 79 and 90.8 Gy_10_ for HR-CTV D_90_, which is consistent with the findings of Hiniker et al. (2013). These results are within the 85—95 Gy HR-CTV D_90_ dose range reported in the EMBRACE II study (Pötter et al. 2018).

The combination of external and internal radiotherapy techniques for advanced cervical cancer demonstrated that a high dose can be administered to the tumour volume while keeping doses to OARs within accepted limits. With CTV_HR_ doses above the ICRU 89 recommendations, high tumour control was achieved, and at the same time the organs at risk received doses below the recommended limits. Comparing the results of the present study with those reported in the literature, both tumour dose and dose to OARs were within similar margins, and better results were obtained for a number of parameters. Thus, brachytherapy can provide a significant boost to increase the dose delivered to the tumour with adequate protection of surrounding organs.

To sum up, the present study has shown that IMRT and VMAT offer a more conformal and homogeneous tumour dose distribution than the 3D-CRT technique, leading to a superior protection for the organs at risk. When EBRT techniques are combined with brachytherapy, it was found that the differences among EBRT techniques regarding doses to organs at risk were much smaller, yet the intensity modulated techniques still offered better protection, especially for the intestine.

The present study is not without limitations. For example, a shortcoming is the relatively low number of patients included in the study. However, this number is considered to be still large enough for a statistically significant evaluation of dosimetric differences. This judgement is confirmed by other studies reported in the literature using similarly small patient cohorts (Cozzi et al. 2008; Guy et al. 2016; Lv et al. 2014). Another limitation is that the dosimetric comparison was conducted between real treatment (3D-CRT) vs. simulated treatment plans (IMRT/VMAT), a study which otherwise could not have been conducted on the same patient cohort. Finally, evaluation of any inter-fraction modifications in patient morphology was not part of the present study. Given that changes in target volume morphology may occur due to organ filling, bowel peristalsis, and setup errors, these effects are likely to reduce the dosimetric advantage of IMRT reported in this work.

It is also to be noted that there is a hypothetical premise in the dose superposition of EBRT and BT, which is that the location of high-dose regions in OARs coincides. As this assumption is not always reached in all cases and for all OARs, the final dosimetry is expected to be affected when the high-dose region in OARs from external beam planning is not located in the same position as its counterpart in brachytherapy planning.

## Conclusions

Generally, for the treatment of cervical cancer intensity-modulated techniques such as IMRT and VMAT show dosimetric advantages over the traditional 3D techniques such as 3D-CRT. In addition to providing better compliance and homogeneity, intensity-modulated techniques provide superior protection for OARs, especially for bowel bag. From the results obtained in the present study it is concluded that combined brachytherapy and intensity-modulated techniques provide the best protection for OARs, particularly for the intestine. While the present results suggest advantages of intensity modulation over 3D-CRT, the dosimetric differences between those techniques are reduced when combined with brachytherapy.

## Data Availability

The data that support the findings of this study are available from the corresponding author upon reasonable request.
